# Impacts of building information modelling (BIM) on communication network of the construction project: A social capital perspective

**DOI:** 10.1371/journal.pone.0275833

**Published:** 2022-10-11

**Authors:** Yao Huang, Lufeng Wu, Jindao Chen, Hao Lu, Jiajun Xiang

**Affiliations:** 1 Key Laboratory of Highway Engineering of Ministry of Education, School of Traffic & Transportation Engineering, Changsha University of Science & Technology, Changsha, China; 2 School of Public Affairs, Nanjing University of science and technology, Nanjing, China; 3 School of Civil Engineering & Engineering Management, Guangzhou Maritime University, Guangzhou, China; 4 Center of Information Technology, China Construction Eighth Engineering Division Corp. Ltd (Southwest Branch), Chengdu, China; 5 School of Traffic & Transportation Engineering, Changsha University of Science & Technology, Changsha, China; Northeastern University, UNITED STATES

## Abstract

Building information modelling (BIM) is considered to be significant for organisational communication in construction projects. However, the role of BIM has not been fully shown in practice. To this end, this study examined the impact of BIM on communication network from inter- and intra- organisational relationships in the construction project. First, the structures of the communication networks before and after the use of BIM in a project in China were determined based on the social capital perspective. Then, the social network analysis was adopted to measure the changes in network metrics (i.e., number of ties, density, centrality, centralisation, and clique). Results shows that the connections among nodes are denser, and all the values of the network centralisation decrease after BIM application compared with the situation before BIM use. Nevertheless, results also shows that some construction project participants, who originally have interaction and communication needs, remain unable to establish effective connections among one another even after BIM use. Accordingly, some suggestions were proposed to solve the issues and deficiencies. This research contributes to (a) the state of knowledge by proposing social capital perspective that can identify inter- and intra-organizational relationships of the construction project from social interaction and common cognition to build communication network, and (b) the state of practice by identifying conditions and proposing strategies for strengthening organisational communication and collaboration in BIM-enabled network relationships.

## 1. Introduction

A construction project has the characteristics of temporary and openness, and the project team often involves numerous participants consisting of different departments in various organisations [1]. The complexities of the project itself and its organisational structure severely deepen the obstruction of information communication. This case can lead to a series of construction issues (e.g., design changes, re-work, quality defects, project delays, cost overruns, and safety incidents), which will seriously affect the project’s performance [2]. Therefore, the efficiency of communication among team members is essential in achieving the goal of the construction project performance. However, in traditional project management, information integration and real-time exchange to achieve efficient communication and collaboration among multidisciplinary team members remain challenging [3]. Fortunately, the advent of building information modelling (BIM) offers an opportunity to address these issues more effectively in the architecture, engineering, and construction (AEC) industry [4–6].

BIM is an advanced and revolutionary technology for generating, visualising, and analysing architectural models, thus changing the way buildings are conceived, designed, built and operated [7, 8]. Moreover, BIM is considered a platform that encourages communication and participation of all participants throughout the lifecycle of the construction project, thus enabling the integration of organisations [9]. These are also the prerequisite for the rapid identification of project management problems and the efficient provision of immediate communication and responses in the new generation of construction project management. Thus, BIM is considered to be able to foster novel and creative alternatives to traditional AEC technology as well as organisation communication innovation in construction projects [10]. However, in practice, the effect of BIM has not been fully shown [9, 11].

The construction project team involves different participating organisations and different departments within each organisation. Both internal and external communication activities exist in a construction project. Internal communication activities within an organisation are often known with fewer tasks compared with external communication, and their importance is often overlooked [12]. Effective internal and external communication by all participants is required for a project’s goal achievement [1]. The impact of BIM on organisational communication in a construction project may be able to be shown by the structure change of communication network that integrates inter- and intra- organisational relationships when BIM use.

Thus, this study introduces the concept of social capital (SC) to reflect the inter- and intra-organisational network relationship of the project, and SC involves social interaction and common cognition dimensions [1]. Bridging SC is contained in links between inter-organisational members from different social characteristics, whereas bonding SC is contained in connections amongst intra-organisational members with the same backgrounds [1]. Therefore, the organisational relationships within a construction project can be treated as a combination of external and internal interactions and consensus. Based on this, the organisational communication networks of the construction project before and after the use of BIM were determined.

A more explicit, objective, and direct measure of the organizational communication network changes attributed to BIM implications will facilitate quantifiable and empirically validated BIM impact assessment and further support BIM implementation in the industry. In addition, previous studies have found that social network analysis (SNA) is an effective tool for measuring metrics of the communication network [13], which is used to quantify the SC of a communication network [14].

The main research questions of this study are: (1) Is there a difference between organizational communication networks of the construction project before and after BIM use? (2) If so, how does the difference between before and after BIM use inspire a more effective project management means and organizational communication mode changes? To answer these questions, this study first determined the organisational communication network before and after the BIM use in a project in China based on the social capital perspective. The SNA was then adopted to measure the changes in network metrics. This research contributes to (a) the state of knowledge by proposing social capital perspective that can identify inter- and intra-organizational relationships of the construction project from social interaction and common cognition to build communication network, and (b) the state of practice by identifying conditions and proposing strategies for strengthening organisational communication and collaboration in BIM-enabled network relationships.

The remainder of this paper is organised as follows. The next section reviews the related works and elaborates on the existing research gap. Then, good empirical data generated from a BIM construction project in China will be analysed using SNA. Finally, the study’s findings will be presented and discussed.

## 2. Literature review

### 2.1. Organisational communication and BIM

For an organisation, communication is considered the base of its operation [12]. Communication plays a huge role in maintaining organisational relationships [15]. In the field of management, poor communication can lead to negative results that reduce organisational performance, whereas effective communication is an important factor affecting the success of an organisation. For effective communication in the organisation, two types of communication, namely, inter- and intra-organisational communication, are required simultaneously [12]. Inter-organisational communication is an external communication that usually involves stakeholders, suppliers, institutions, customers, and others. Then, intra-organisational communication is internal communication, usually involving various departments in the organisation.

As a temporary social system, a construction project involves the interaction of a group of numerous professionals from different disciplines in its prolonged implementation process, resulting in the complexity of the project’s organisational structure and relationships [1]. Effective construction project management requires the establishment of an integrated organisational communication network, so as to realise adequate information communication and sharing across the whole project lifecycle [16, 17]. Under these circumstances, BIM has emerged as a representative construction information and communication technology (ICT), considered by professionals to achieve better communication and collaboration [1, 18]. Integration of BIM and digital technologies, such as immersive technologies [19], artificial Intelligence [20], digital twin [21], etc., shows great potential in the promotion of construction management innovation. Theoretically, building-related information can be effectively integrated within advanced digital technologies in a BIM-enabled environment [9].

On the one hand, previous studies have provided valuable references for establishing the relationship between BIM implementation and improved organisational communication and collaboration from technological standpoint [22, 23]. However, in practice, the effect of BIM-supported communication and collaboration has not yet been fully shown [9, 11]. For this reason, it has become increasingly important to analyse the impact of BIM on the construction project from an organisational and network perspective with the rapid development and adoption of BIM.

On the other hand, research on the combination of inter- and intra-organisational linkages amongst all participants of a construction project under the BIM influence remains limited. Similarly, in practice, internal communication activities are often known to have fewer tasks compared with external communication, and most managers do not realise the importance of internal communication within an organisation [12].

Notably, a significant part of the management literature discussed communication about external and internal organisations at the company level. For example, Campbell and Alexandra [24] regarded that how members within the firm interact or communicate with external members is determined by recognised social norms or norms that have been institutionalised within the firm. Othman et al. [25] believed that strengthening relationships with key stakeholders based on good intra-organisational communication will increase corporate profits and business growth. Furthermore, Liu [26] proposed that an interaction exists between the structure of external and internal communication networks. This notion suggests that internal cooperative norms will affect the external relationships of an institution. Similar views can be obtained in the literature of organisational cooperation and strategic alliances [27].

Previous studies showed that external and internal communications are closely related at the company level. Insight into the nature of this connection will help managers improve their efforts by designing effective external and internal relationships. Similarly, the ad hoc organisational network relationships within a construction project are a combination of external and internal interactions, including consensus connections [1]. Thus, at the project level, the simultaneous integration of inter- and intra-organisational communication relationships should also be investigated, particularly for construction projects under the influence of BIM.

### 2.2. Organization level SC

Social capital is defined as the sum of embedded, available, and derived resources in a network of social relationships owned by an individual or organisation [28]. The network resources include not only the channels where individuals can obtain information, but also the network location and status of individuals in the context of specific actions [14, 29]. As an intangible asset contained in the social network, SC could promote certain behaviours of individuals in a specific network [30]. SC can be divided into three dimensions, namely, structural, cognitive and relational dimensions [31]. The structural dimension of SC consists of social interaction, appropriate organisation, network ties and network configuration [32, 33]. These components form the overall connection model amongst group members. Cognitive SC, which comprises a common understanding amongst network members, encourages individuals to communicate with one another as they share the same language and vision [34]. Relational SC includes resources and information in interpersonal networks, such as identity, trust and norm [35].

Organization level team SC is a special type of SC in which the relations of team members are formed through formal and informal structures [36, 37]. A temporary project team consisting of different participants aims to achieve a clear shared goal [38]. Achieving the goals of a project often requires a diversity of creative knowledge and ideas that is beyond the capacity of a single member. SC is often seen as an important source of innovation, because it promotes knowledge sharing amongst team members [39]. This indicates that the enhancement of team SC is important to a project’s success to some extent. The team SC of a project is a combination of internal relationships within a collective (i.e. bonding SC) and external relationships amongst different groups (i.e. bridging SC) [1, 40]. Bonding SC consists of the linkages amongst members with the same backgrounds, whilst bridging SC connects organisations or groups from social characteristics [41].

There is no doubt that a construction project team consists of many participants (i.e. organisations and departments within these organisations) and that the characteristics of bonding and bridging SC are reflected in it [1]. According to the characteristics of a construction project team, Huang et al. [1] clarified that SC within an organisation is bonding SC, whereas the SC between different organisations is bridging SC. Both bonding and bridging SC can be operationalised structural, cognitive, and relational dimensions. Specifically, the structural dimension is defined as social interaction or ties [42], cognitive dimension is operationalised as a shared vision or ‘same language’ [34] and the relational dimension is defined as the norm, trust, or commitment [43].

### 2.3. BIM and organization level SC

Some research believed that ICT could influence the SC in a team to some extent [1]. The popularity of ICT is changing normative structures and relationships within social networks [44]. ICT can provide frequent interactions amongst individuals or organisations. Such interactions amongst different members can help strengthen understanding and trust whilst simultaneously creating a shared vision or consensus amongst the members [31]. As a construction ICT, BIM is considered to be applied across the whole lifecycle of the construction project, encouraging fast and full information sharing by bringing together the work of different disciplines and guided by the aim of improving collaboration amongst stakeholders [45]. By using a centralised model, collaborations amongst team members can be increased, which is a widely publicised advantage of BIM [46]. Given that information access and communications can be improved in this way, the extent of work carried out in isolation can also be reduced. Theoretically, the application of BIM can inevitably affect the change of team SC in a construction project.

For the SC structural dimension, BIM may stimulate more intensive and higher dependent interactions amongst participants by changing the efficiency and means of information exchange, thereby influencing the collaborative process [1]. According to Alizadehsalehi et al. [23], BIM facilitates intelligent and real-time interaction between individuals or between individuals and data/information in the AEC industry. For the SC cognitive dimension, a critical problem with AEC team collaboration is that it is difficult to form a mutual understanding and common project vision amongst all the participants due to the temporary and fragmented nature of the construction project [47]. Different participants generally tend to focus on and optimise their own work [48]. Hence, some researchers explored the relationship of BIM and the enhancement of consensus and collaboration amongst the project team. For example, Forgues et al. [48] believed that BIM plays a critical role in promoting the information fusion to reach a mutual understanding of the overall process information flows amongst stakeholders. Meanwhile, Dounas et al. [49] proposed a BIM-based consensus mechanism to navigate issues of trust, transparency, and consensus between design agents. For the SC relational dimension, the grounded analysis indicates that experience, especially the application of BIM, may promote the trust development in a project team [50]. Liu et al. [51] also believed that communication through BIM has a positive impact on the formation of trust amongst a project team.

Furthermore, as a network variable, SC reflects the degree of control over resources (information) by actors within a social network [1]. Many possible measures, such as size, density, cohesion, and closeness of social networks, are candidates for SC and drivers of practical outcomes at work [14]. Therefore, the BIM based communication network relationships within a project can be determined by the SC of participants. Then, the values of network metrics can be measured by social network analysis (SNA). By combining SC theory and SNA, knowledge transfer, communications, and sense of trust amongst various participants of a construction project can be analysed deeply [52].

Inspired by this, the current paper aims to provide additional evidence regarding the impacts of BIM on both inter-and intra-organisational network relationships for all participants of a construction project based on SC theory.

## 3. Research methodology

This study was carried out in strict accordance with the recommendations and guidelines on Human Subject Research (involving human participants and/or tissue) for scientific purposes, and this study was approved by the Institutional Human Subject Research Committee, China (Protocol Number: CSCUST-DW-2020-075). This research involves Human Subject Research (i.e., expert interview). However, the data in this paper has been collected and analysed anonymously. We ensured the respondents that the survey has been conducted only for academic purposes, and there is no personal identification. The data of expert interview have obtained the written consent of the participants. The interview consent is in the supporting information uploaded by the system.

**Fig 1:**
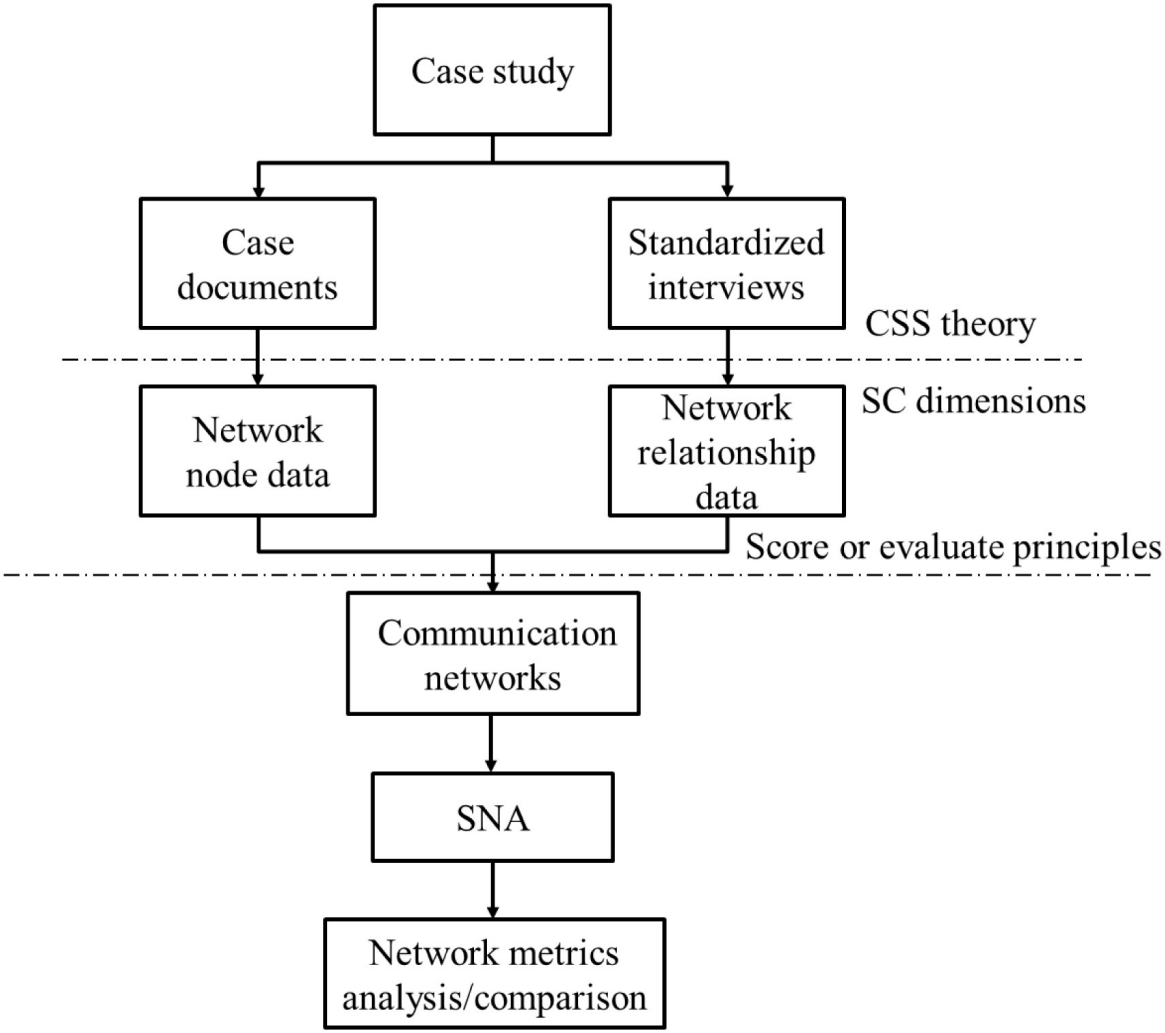
Research process.

The overall research design is a case study, with SNA at the kernel. Firstly, this study determines the communication network relationships of a project team before and after BIM use from the dimensions of SC. Then, this work measures the changes of network metrics using SNA, and the differences between the two networks were compared. Fig 1 shows the research process. Generally, a comparative study had better select two or more similar cases to investigate the impacts of a particular intervention [53]. However, the environments or teams between two construction projects can rarely be considered identical. Finding two projects (one that utilised BIM and one that did not) with exactly the same characteristic for comparison is difficult. Hence, this study only focuses on one project but collects twice data through case documents and interviews with main project actors to build corresponding communication networks.

Amongst them, the collection of interview data was mainly based on the ‘self-perceptions’ (combining personal experience and own understanding) of interviewees to judge the relational changes of communication network before and after BIM use. The theoretical basis of ‘self-perception’ here is cognitive social structure (CSS) theory proposed by Krackhardt [54]. This is a kind of thinking mode of relational theory that also belongs to the research category of social network. CSS studies the combination of formal and informal relationships.

### 3.1. Case study

The case study method is the most appropriate for this research, as the researchers sought to establish rich and empirical descriptions of project team communication in a context where researchers do not interfere [53].

To make the case representative, the selected case must meet the following criteria: 1) the organisational structure of the construction project selected must be well-defined and the case data obtained must be relatively detailed and complete, and 2) the construction project selected as the case study should have a high level of BIM application, and each participant in the selected case project has a deep understanding of the BIM. The selected BIM project was in a city of China. This project was the first phase of a city commercial complex, covering a gross site area of 6,489.39 m^2^ and a total construction area of 66,872 m^2^. This project had three floors underground and 23 floors above ground, with a total investment of RMB 280 million and a total construction period of 29 months. BIM had been used in the whole process management of this project.

Participating units in this project included the owner, the designer, the engineering supervision, the BIM consultant, the general contractor, and the subcontractor. The main departments established by the project owner included the engineering, design, operations, and cost department, among others. Meanwhile, the main departments set up by the contractor include the engineering, technology, cost, safety, and material departments. Although several subcontractors participated in the project, only one subcontractor most relevant to BIM use was selected as the representative in this study. The main departments set up by this subcontractor were the engineering and cost department. In addition, the engineering supervision, the designer, and the BIM consultant in the project had no other sub-departments.

### 3.2. Structured interview

Apart from the collection of case documents, the structured interview for the main project participants is also another complementary way to obtain data [55]. The structured interview has the potential to overcome the poor response rates of a questionnaire survey [56]. Compared with the questionnaire survey and unstructured interview, the structured interview has better reliability and validity [57, 58]. In this interview, the self-perceptions of interviewees are the basis to obtain the relationship data of communication network before and after BIM use.

According to SC theory, the sense of trust among participants is the subjective condition of information communication, while social interaction and common cognition among them are the objective basis of communication [1]. Trust can be improved by social interaction and common cognition. Therefore, in this study, the criterion for determining the existence of information communication relationships is whether interviewees believed that interaction needs and consensus with others exist. The outline of the interview questions contains two dimensions of social interaction and common cognition in this paper. All interview questions/questionnaire were determined or developed based on existing studies, e.g., Levin and Cross [59], Lin [60], Hau et al. [61], and Tsai et al. [32]. There may be some interference or redundant items (interference variables) in the questionnaire [62]. Therefore, the reliability and validity of the questionnaire should be analyzed, and the questionnaire should be further optimized.

Firstly, the category analysis method was used to divide the original interview questions into blocks and form two dimensions (i.e., social interaction and common cognition). The content analysis method was then used to find the useful information and items from the two dimensions. Then, the semi-structured interviews with experts were conducted to assess the appropriateness of the questions’ scope and avoid ambiguous expressions. All five experts who used the semi-structured interview had more than 20 years of practical or research experience in the AEC domain and are also familiar with BIM application in construction projects. Two of them are project managers of major projects, two are university professors, and another is a senior manager of an architecture firm. Through the semi-structured interviews, redundant items were merged, and missing items were added, and all interview questions were modified to suit the context of the AEC industry. Subsequently, a pilot questionnaire survey involving 166 respondents (who have at least 3 years of practical experience in the construction domain) as an exploratory factor analysis of all responses was carried out to verify whether all of the questions were classified into their corresponding dimensions (i.e., social interaction and common cognition). Based on the feedback, the questionnaire was further optimized, and the final questionnaire was formed. Ultimately, the final questionnaire was adjusted appropriately to be the standardise interview outline (S1 and S2 Files).

As it was necessary to compare the communication networks before and after the application of BIM, two interview outlines were made for the same interviewees from the selected case project. One was about the self-perceived information communication relationships of interviewees before BIM use, in which the BIM consultation party was excluded in the network nodes. The other was about the self-perceived information communication relationships of interviewees after BIM use, in which the BIM consultation party was included in the network nodes.

### 3.3. SNA

Social network analysis (SNA) is defined as a tool for analysing a set of related actors or parties who either cooperate or compete with one another through social structures [63]. It provides researchers with a wide range of possible measures of network properties related to underlying characteristics of the network and thus presents several ways of operationalizing structural embeddedness. The approaches used to investigate project networks can be qualitative or quantitative. SNA uses a collection of graphs and other mathematical models analyse useful in social sciences, economics, political science, computer science and others to help analyse social network attributes related to roles, connections, interactions, and metrics [63]. SNA can help in visualising the changing relational patterns amongst project actors [64]. Moreover, SNA focuses on the structures and patterns of relationships over time, with the aim of examining how relationship structures affect behaviours and determine the causes of such behaviours [13, 65].

The wide range of network metrics in SNA includes the number of ties, network density, average path length, actor centrality, centralisation, clique, average clustering coefficient and modularity, among others [32, 63, 65]. Amongst them, number of ties refers to the number of relations contained in a network. Network density is related to the overall network structure. Path length and actor centrality gauge the distance between nodes, whilst centralisation (e.g., degree, closeness and betweenness centralisation) refers to the differences of the corresponding centrality of network actors. Clique is a subset of actors who are more strongly connected than they are with other actors who are not part of the group. Furthermore, modularity and average clustering coefficient describe the flexibility of clusters. These metrics represent the standings and relationships of the nodes, which can be used to analyse the flexibility of networks and reflect communication efficiency [63, 64].

### 3.4. Network data collection

The premise of establishing the communication network of a construction project team is to collect the relevant node data and relationship data of the network. Firstly, the specific participants were identified according to the documents pertaining to the organisational structure of the selected case project and used as the nodes of communication network. In this case, the network nodes were as follows: owner’s engineering department (OED), owner’s design department (ODD), owner’s operation department (OOD), owner’s cost department (OCD), designer (Des), engineering supervision (Sup), BIM consultation (BC), contractor’s engineering department (CED), contractor’s technology department (CTD), contractor’s cost department (CCD), contractor’s material department (CMD), contractor’s safety department (CSD), subcontractor’s engineering department (SED) and subcontractor’s cost department (SCD).

Furthermore, the structured interviews were adopted to collect the relationship data amongst the network nodes before and after BIM use, respectively. This study selected the main managers or backbone employees from each participant (i.e., network node) as the representative of the interviewees. All the interviewees had more than 10 years of experience in the AEC industry and over 5 years of learning/practical experience in BIM. An overview of interviewees is in S4 File. The interview guidelines were sent to all interviewees at least two days before the interviews so that they can be fully prepared. During the process of the interview, firstly, an introduction for the interview was given, and the critical questions were explained by interviewers, to form a common understanding among interviewees. Afterwards, the interviewees were asked how BIM is used in their department. Finally, they answered the questions of interview outline one by one. The answers of the interviewees were counted to score the social interaction and common cognition dimensions of the outline. Accordingly, whether an information communication relationship (connection) exists between project participants (nodes) can be evaluated. The principles to score and evaluate the interactive and cognitive relationships including information communication relationships between different participants are as follows.

For questions of social interaction dimension, if interviewees A and B mentioned each other in more than half of the total number of questions, then we considered that A and B had an interaction relationship. In this case, their social interaction dimension was given a score of 1. Otherwise, if interviewee A or B mentioned each other less frequently, then we considered that A and B had no interaction relationship. In this case, their social interaction dimension was given a score of 0. For example, the interview for the representative of the OCD revealed that this person mentioned the CCD four times when answering the social interaction questions, and vice versa. The number is greater than half (2.5) of the total (5) number of questions. Therefore, an interaction may exist between these two departments, and their score of the social interaction dimension is 1. However, the two nodes are only verified to have a connection when both the social interaction and common cognition dimensions have a score of 1. In this case, the corresponding value in the final relationship matrix was set to 1. Otherwise, the corresponding value in the final relationship matrix was set to 0. The 0–1 matrixes of communication relationships before and after BIM use are shown in Tables 1 and 2, respectively.

**Table 1:** 0–1 matrix of communication relationships before BIM use.

Participants	OED	OCD	OOD	ODD	CED	CTD	CCD	CSD	CMD	SED	SCD	Sup	Des
OED	0	1	1	1	1	1	0	0	0	0	0	1	1
OCD	1	0	1	0	0	0	1	0	0	0	0	1	1
OOD	1	1	0	1	0	0	0	0	0	0	0	1	0
ODD	1	0	1	0	1	1	0	0	0	0	0	1	1
CED	1	0	0	1	0	1	1	1	0	1	0	1	1
CTD	1	0	0	1	1	0	1	1	1	0	0	0	1
CCD	0	1	0	0	1	1	0	1	0	0	1	0	0
CSD	0	0	0	0	1	1	1	0	1	0	0	0	0
CMD	0	0	0	0	0	1	0	1	0	0	0	0	0
SED	0	0	0	0	1	0	0	0	0	0	1	0	0
SCD	0	0	0	0	0	0	1	0	0	1	0	0	0
Sup	1	1	1	1	1	0	0	0	0	0	0	0	0
Des	1	1	0	1	1	1	0	0	0	0	0	0	0

**Table 2:** 0–1 matrix of communication relationships after BIM use.

Participants	OED	OCD	OOD	ODD	CED	CTD	CCD	CSD	CMD	SED	SCD	Sup	Des	**BC**
OED	0	1	1	1	1	1	1	0	0	0	0	1	1	1
OCD	1	0	1	1	0	0	1	0	0	0	0	1	1	1
OOD	1	1	0	1	0	0	0	0	0	0	0	1	0	0
ODD	1	1	1	0	1	1	1	0	0	0	0	1	1	1
CED	1	0	0	1	0	1	1	1	0	1	0	1	1	1
CTD	1	0	0	1	1	0	1	1	1	1	0	1	1	1
CCD	1	1	0	1	1	1	0	1	0	0	1	1	0	1
CSD	0	0	0	0	1	1	1	0	1	0	0	0	0	0
CMD	0	0	0	0	0	1	0	1	0	0	0	0	0	1
SED	0	0	0	0	1	1	0	0	0	0	1	0	0	1
SCD	0	0	0	0	0	0	1	0	0	1	0	0	0	0
Sup	1	1	1	1	1	1	1	0	0	0	0	0	0	1
Des	1	1	0	1	1	1	0	0	0	0	0	0	0	1
**BC**	1	1	0	1	1	1	1	0	1	1	0	1	1	0

We observed that the scores of social interaction and common cognition between the OCD and the CCD before BIM use are both 1. Therefore, there is an effective information communication between these two parties and a communication line is formed between the corresponding two nodes in the communication network. This point is proven in practical engineering, in which both objective interaction needs (business contact) and subjective common cognition (the same technical language, etc.) exists between the OCD and the CCD. In comparison, the score of social interaction between the OCD and the SCD is 0, whilst the score of the common cognition between them is 1. Therefore, there is no effective information communication relationship between the two parties. Specifically, there is no connection between the two nodes in the communication network. This point is also proven in practical engineering: although the OCD shares the same technical language with the SCD, they have no direct business contact with each other during the implementation process of the case study project.

## 4. Data analysis and results

**Fig 2:**
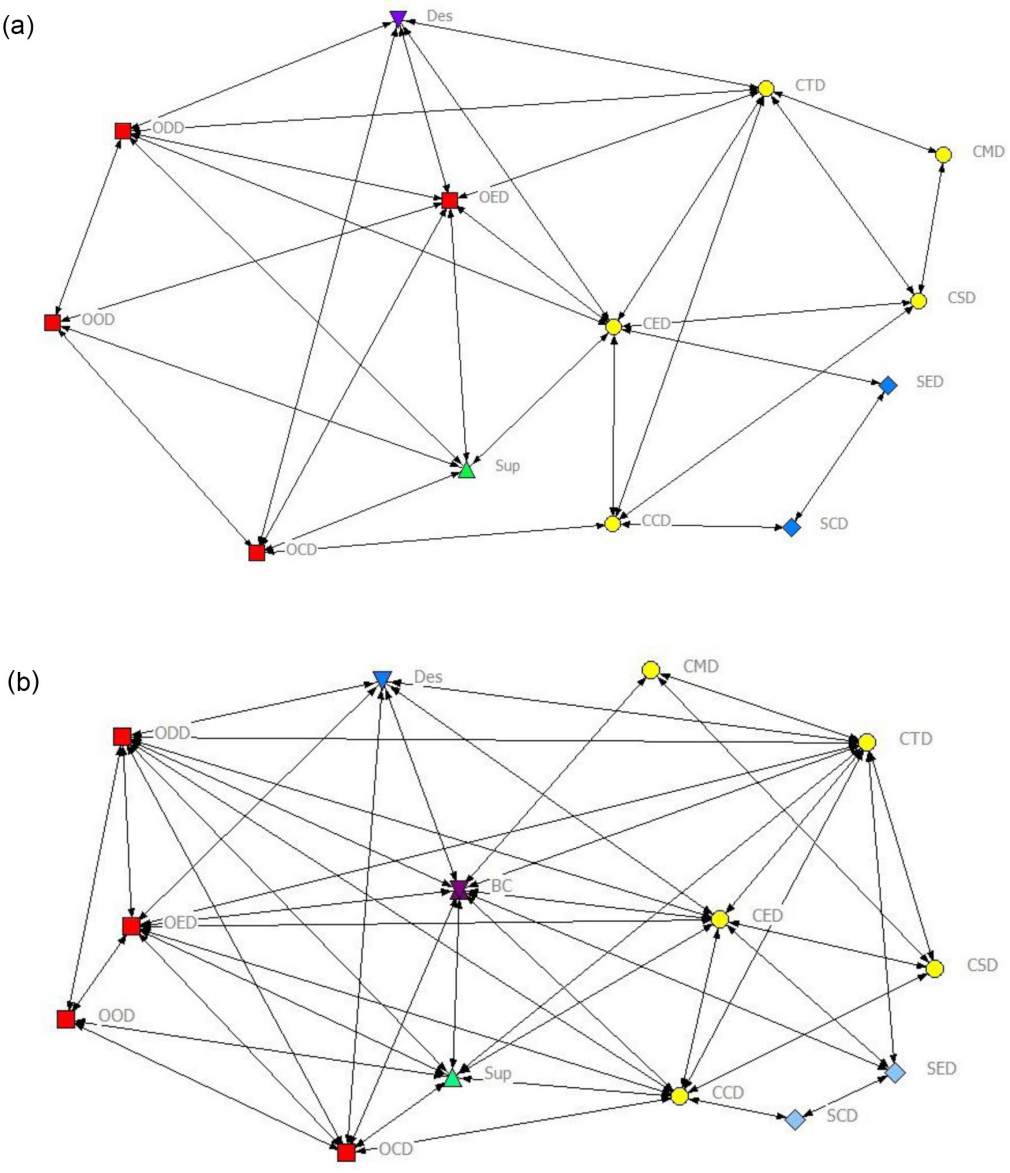
Communication networks of the project team. (a) Before BIM use. (b) After BIM use.

Two 0–1 matrixes of information communication relationship were inputted into NetDraw software to form a corresponding graphic of communication networks of the project team before and after BIM intervention, as shown in Fig 2(a) and 2(b), respectively. The nodes comprise project participants (actors), and the links show the relationships between them in these networks. Meanwhile, Ucinet6.0 software was used to analyse the network density, number of ties, average path length, centrality, centralisation, and clique before and after BIM use.

Obviously, the connections amongst participants in the communication network after BIM use became denser and closer than the situation before BIM use.

### 4.1. Network cohesion metrics

The overall network density and number of ties represent the degree of connection between network members [66]. Table 4 shows that the value of network density is 0.3974 before BIM use, which is less than 0.5, indicating a low level of network density [67]. On the contrary, the value of network density was 0.5165 (>5) after BIM use. Meanwhile, the values of the number of network ties in the situation before and after BIM use are 62 and 94, respectively. The path length is the average of the shortest paths between all pairs of nodes [68]. Table 3 shows that the average path length of the network after BIM use is 1.549, which is slightly lower than that before BIM use (1.769).

**Table 3:** Network cohesion metrics analysis results.

Metrics	Before BIM use	After BIM use
Network density	0.3974	0.5165
No. of ties	62	94
Avg.path length	1.769	1.549

The analysis results of density, number of ties and average path length suggest that the connections amongst participants in the communication network after BIM use became denser and closer than the situation before BIM use. According to Pryke [64] and Lusher et al. [66], the greater the network density and number of ties, the closer the connection between network members; the network with short path length is considered efficient networks by which information travels fast. This finding indicates that the introduction of BIM can shorten the original path of information transmission or build more new paths and help to improve the communication efficiency between participants.

### 4.2. Network centrality metrics

Centrality is an important metric to measure the degree of network centrality of a node in social network analysis, which reflects the power, status, and importance of a node in the network [63, 64]. The centrality analysis mainly contains degree centrality, closeness centrality, betweenness centrality analysis and others [32, 64].

**Fig 3:**
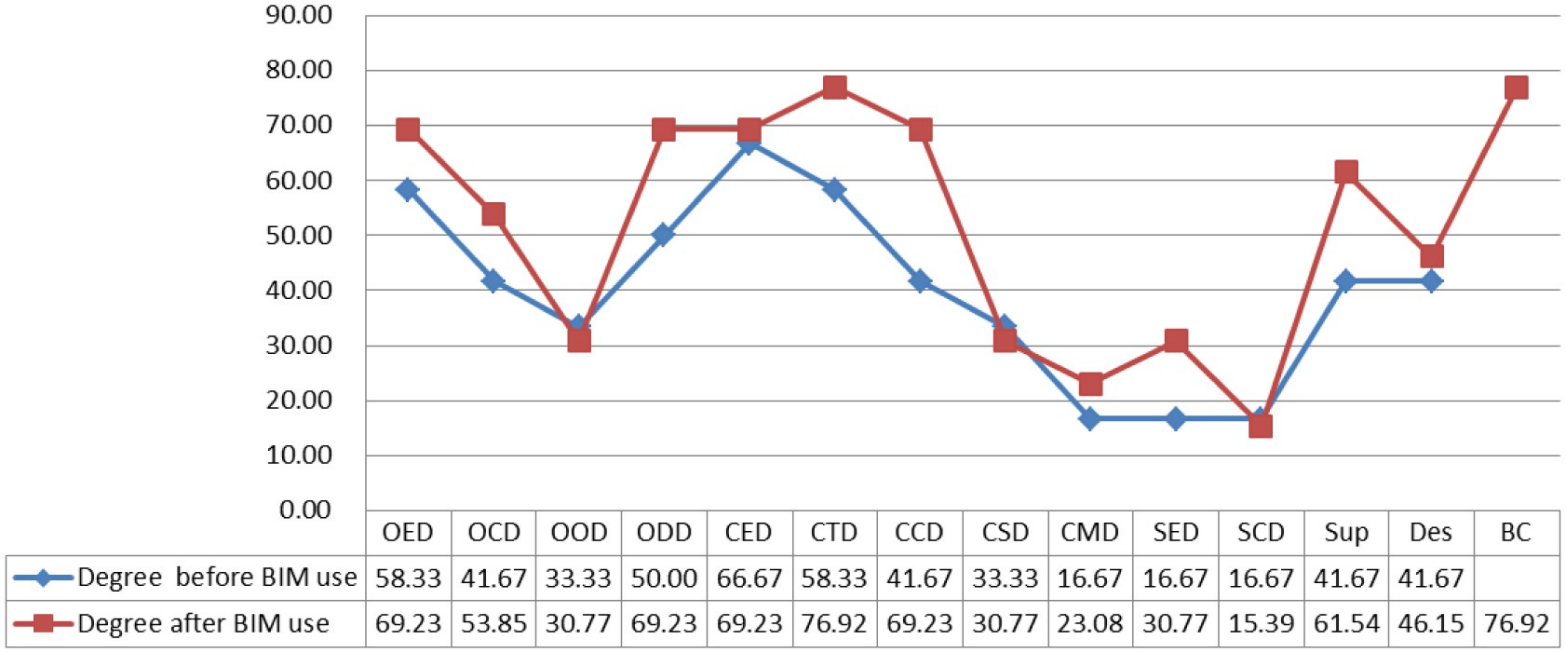
The degree centrality in project communication networks.

**Fig 4:**
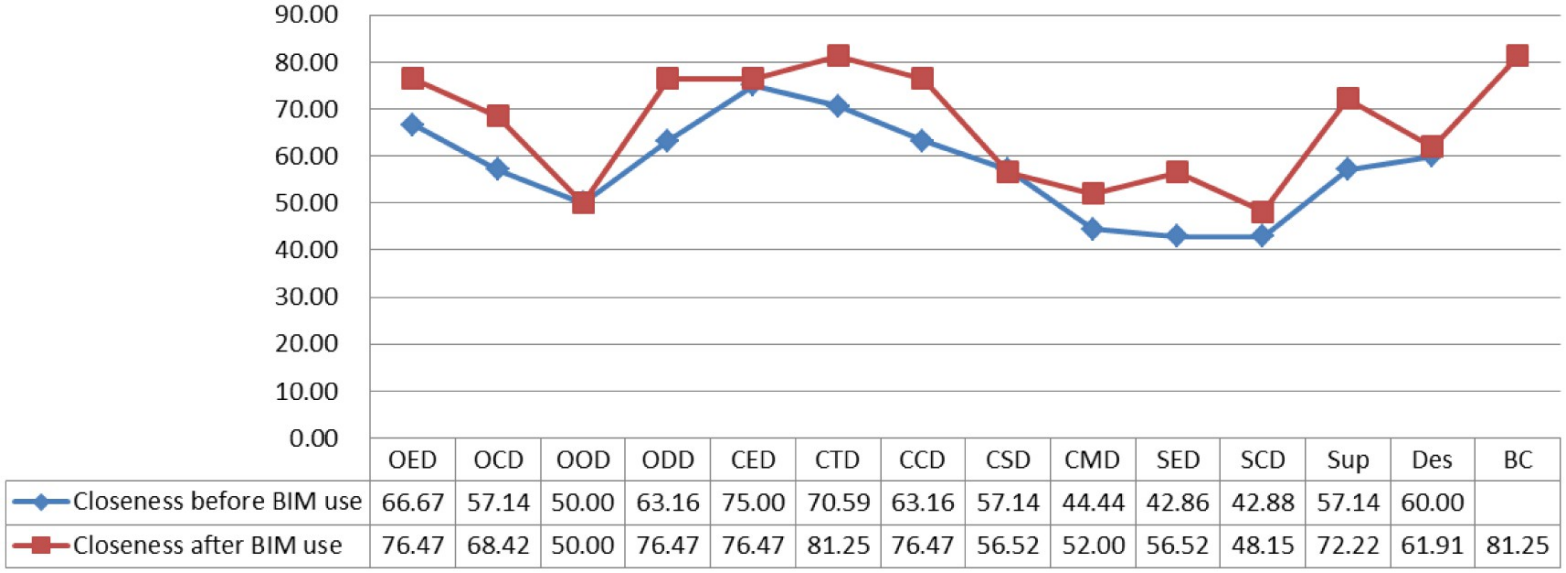
The closeness centrality in project communication networks.

Degree centrality refers to the number of neighbouring nodes directly connected to a node, whereas closeness centrality reflects the degree of proximity between a node with others in the network [63, 64]. Figs 3 and 4 depict the changes of degree and closeness centrality in the project communication networks between before and after BIM use, respectively. The degree and closeness centrality of most nodes (OED, OCD, ODD, CTD, CCD, SED, and Sup) have evidently increased after BIM use compared with that before BIM use.

**Fig 5:**
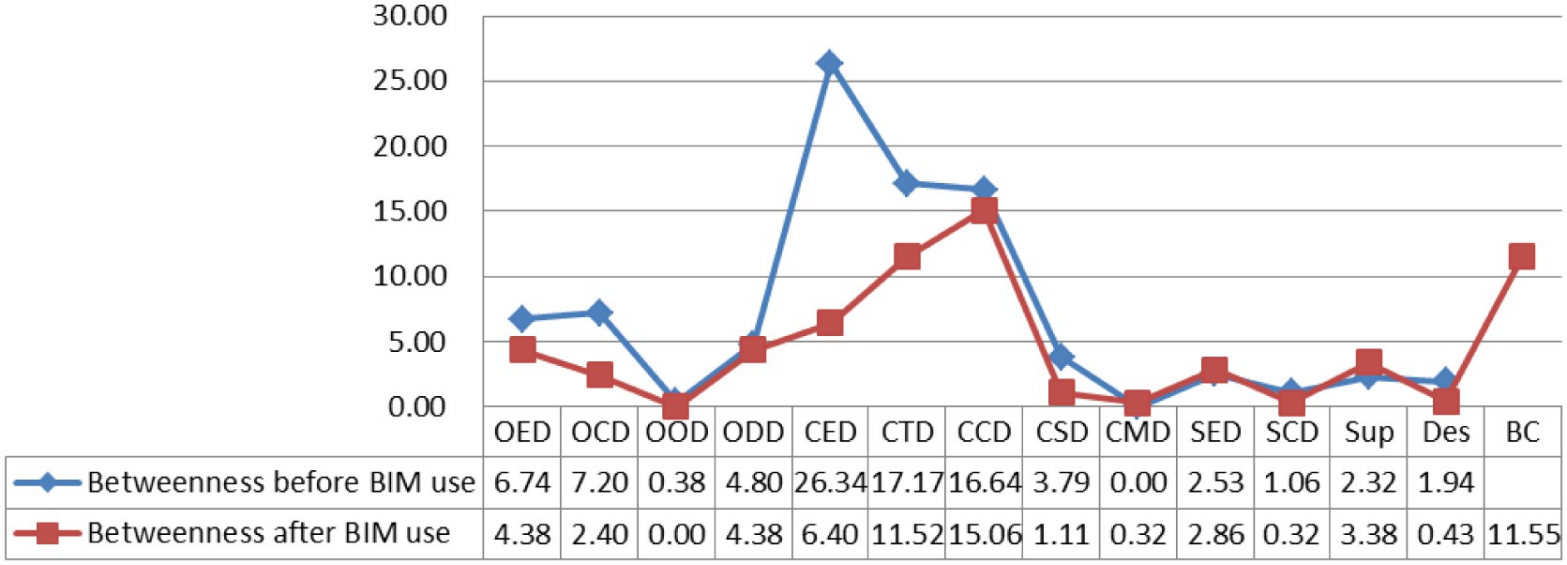
The betweenness centrality in project communication networks.

This betweenness centrality denotes actors who are in the network in a privileged position where, because of its location, they bridge between others, that is, this is the actor that one actor must go through to achieve communication with each other [67]. Fig 5 shows the changes of betweenness centrality in the project communication network between before and after BIM use. The betweenness centrality of some nodes showed a decreasing trend after BIM use compared with the situation before BIM use. In particular, the value of CED has the most significant decrease.

The analysis results of centrality indicate that the degree and closeness centrality of most nodes increased after BIM use. Pryke [64] and Tai et al. [69] noted that the greater the degree of centrality, the more the other nodes connected to this node, indicating the higher the importance of this node. Moreover, the greater the closeness centrality of a node, the closer it is to others. Hence, this finding also suggests that BIM can enhance the number of neighbouring nodes directly connected to a node. However, the betweenness centrality of some nodes decreased after BIM use. According to Borgatti et al. [67], the greater the value of the betweenness centrality, the more resources the node has on the network. This notion, in turn, means that BIM eliminates the control over information by some ‘intermediaries’. Therefore, although the application of BIM reduces the indirect transmission channels of information, the direct transmission channels of information greatly increase. This result reveals the reason why the betweenness centrality of CED in the network decreases significantly after the application of BIM. Before the application of BIM, CED has the highest degree of betweenness centrality, that is, many nodes communicate through it. However, after the application of BIM, more direct links are established between other nodes, so the media role of CED is greatly reduced.

### 4.3. Network centralisation metrics

**Fig 6:**
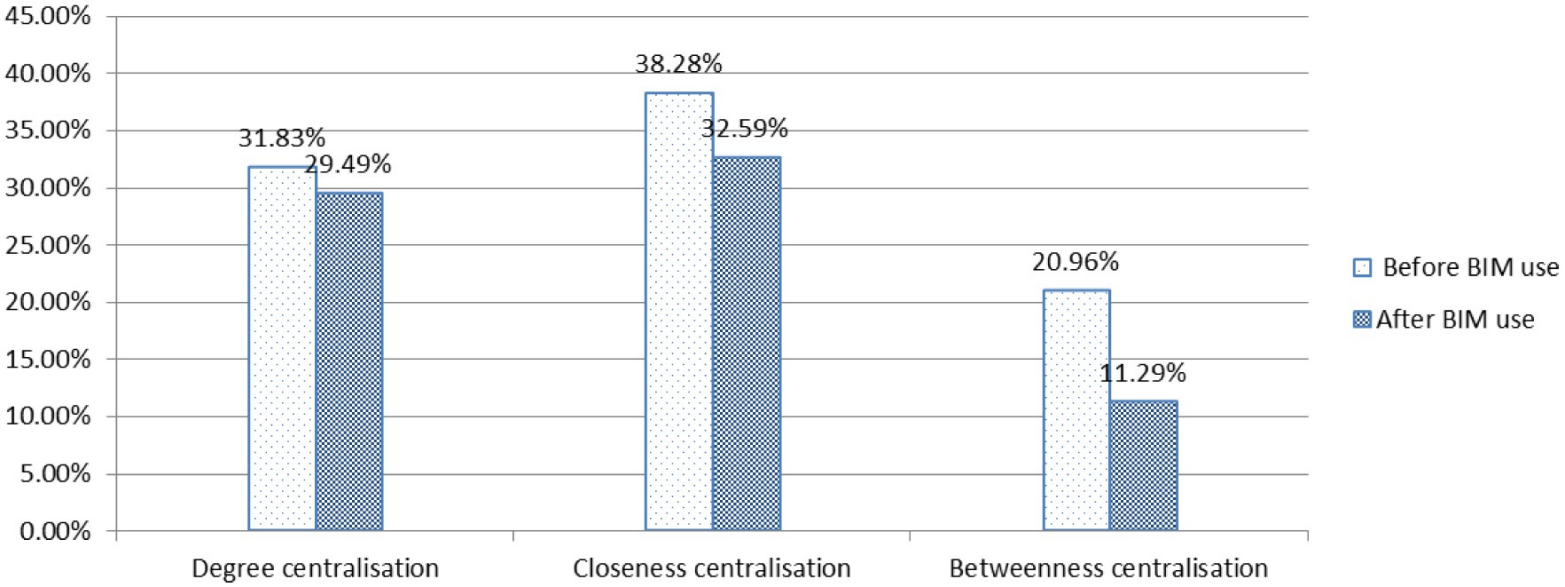
The centralisation of project communication networks.

Network centralisation metrics, such as degree, closeness and betweenness centralisation, are mainly used to represent the different levels of the corresponding centrality of each member in a network [67]. Fig 6 shows the pairwise comparison of centralisation metric values between before and after BIM use. The centralisation metric values of degree, closeness and betweenness after BIM use are 29.49%, 32.59% and 11.29%, respectively, and all of them decreased compared with the situation before BIM application.

Compared with the situation before BIM application, all values of network centralisation of degree, closeness and betweenness decreased after BIM use. According to Borgatti et al. [67], the smaller the value, the more even the centrality distribution of each member and the information distribution in the communication network. This result indicates that the application of BIM resulted in a more balanced information distribution within the communication network. In other words, the degree of information aggregation becomes lower in this project team, and the relevant information can also be more easily accessed by the parties involved.

### 4.4. Network subsets

**Fig 7:**
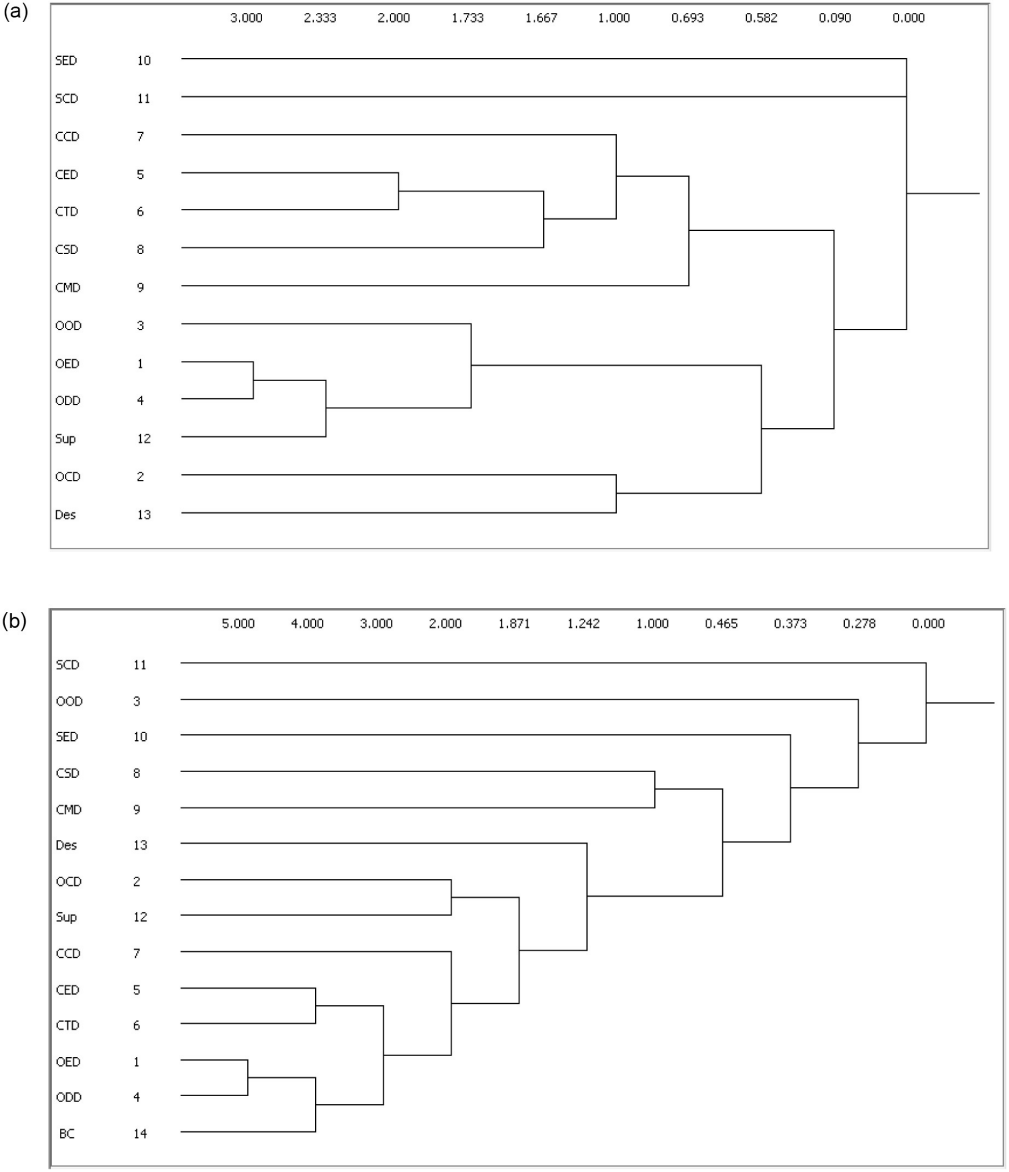
Dendrograms of cliques’ analysis. (a) Before BIM use. (b) After BIM use.

The division of players in subsets or cliques is a very important aspect of the social structure and helps to understand the behaviour of the whole network. A clique is a subset of actors who are more strongly connected than they are with other actors who are not part of the group [70]. To know the extent to which actors are most ‘central’ and most ‘isolated’ from the cliques, and which these substructures overlap, cliques of the project communication networks were examined. Fig 7 and Table 4 show the analysis results. Table 4 illustrates the seven cliques found in the communication network before BIM use, and the nine cliques found in the communication network after BIM use. Amongst them, each clique has a minimum number of three actors.

**Table 4:** Cliques found.

No. of cliques	Before BIM use	After BIM use
1	OED, ODD, CED, CTD, Des	OED, ODD, CED, CTD, CCD, Sup, BC
2	CED, CTD, CCD, CSD	OED, ODD, CED, CTD, Des, BC
3	OED, ODD, CED, Sup	CED, CTD, SED, BC
4	OED, OCD, OOD, Sup	CED, CTD, CCD, CSD
5	OED, OCD, Des	CTD, CSD, CMD
6	OED, OOD, ODD, Sup	CTD, CMD, BC
7	CTD, CSD, CMD	OED, OCD, ODD, CCD, Sup, BC
8	-	OED, OCD, OOD, ODD, Sup
9	-	OED, OCD, ODD, Des, BC

As shown in Fig 7 and Table 4, two completely isolated actors (SED and SCD) are observed in the situation before BIM use; one completely isolated actor (SCD) is observed after BIM use. These actors do not belong to any clique. Moreover, several subsets contain one or more of the same actors. For example, actor CTD simultaneously belongs to three of the seven cliques formed before BIM use. More nodes simultaneously belong to more cliques since the adoption of BIM, but several nodes are still not sufficiently engaged in these cliques. For example, the number of cliques that OOD, CSD and CMD nodes simultaneously belongs to is still smaller than two after BIM use. Thus, in addition to one completely isolated actor SCD, relatively isolated actors OOD, CSD and CMD also exist.

The analysis results of cliques for this project communication network suggest that the number of subgroups involving one or more of the same actors increased after BIM use. Therefore, the application of BIM eliminated a part of the information island, and the diffusion of information resources will become quicker throughout the network.

## 5. Discussion

Before the use of BIM, the results show that the connections amongst nodes are relatively sparse. From the perspective of SC, this phenomenon can be further revealed. Firstly, sufficient interaction and common recognition amongst participants of the ad hoc construction project team are lacking. Thus, these participants are unable to form sufficient trust relationships and communication willingness, resulting in the hampered exchange of information amongst numerous nodes. By contrast, the connections amongst nodes became more intensive, and the information distribution within the network becomes more balanced after BIM use. Combined with the SC perspective, the reason is that the degree of social interaction and common cognition amongst more participants become high after BIM use, thereby fostering an atmosphere that is more conducive to the exchange of information.

The network centralities and connection relations showed that, in addition to the connections between some network nodes and the new participants (i.e., BC) after BIM use, some previously un-contacted participants were also beginning to build communication relationships. All these new connections relate to OED, OCD, ODD, CTD, CCD, SED, and Sup (their degree and closeness centrality evidently increase). The new connections mainly included the following: (a) the ODD with the OCD, (b) the OED with the CCD, (c) the Sup with the CTD and the CCD and (d) the CTD with the SED. The reasons for this may be as follows:

1. The accurate statistics of engineering quantities obtained through BIM can be used to analyse the economic indicators of different design schemes within the budget range [71, 72]. This approach can address the issues where the ODD often prioritises the design effect and tends to ignore the accompanying costs and is not sensitive to costs (OCD).
2. In the process of dealing with engineering changes, the engineering change visa sheets can be associated with the entity model in BIM, thereby facilitating the automatic calculation of the increase or decrease of engineering quantities and costs [73]. This approach can solve the issues where the owner (OED) and engineering supervision (Sup) are prone to dispute with the contractor (CCD) regarding the authenticity and timeliness of the project visa reported.
3. In the process of reviewing the construction scheme, BIM can be used to deepen the scheme design, extract the construction model, simulate the construction scheme of key nodes, and evaluate the rationality or practicability of the construction scheme [7, 8]. Meanwhile, the quality control information of the supervision for key nodes can be loaded in BIM. These processes can address the disputes arising between the Sup and the CTD or between the SED and CTD because of their different understandings of the rationality or practicability of construction schemes for key nodes.

Therefore, with the application of BIM, the task objectives and work processes become more visualised, indexed, and intuitive. In turn, this approach can help form a unified cognition of all parties to a large extent, thereby eliminating some barriers to information exchange and communication amongst participants.

Nevertheless, the subsets analysis results showed that several nodes (i.e., OOD, CSD, CMD and SCD) are still not sufficiently engaged in generated cliques. Thus, the relevant construction project participants who originally had interaction and communication needs were unable to establish effective connections. These failed connections all relate to OOD, CSD, CMD and SCD. For example, no effective connection existed between the following participants: CMD and CED; CMD and CCD; CSD and BC; CSD and Sup; CMD and Sup; OOD and BC; SCD and BC. The reasons for this may be as follows:

1. Even after introducing BIM, the CMD still cannot clearly present or extract the materials, equipment and components related to the engineering entity information to the CED, CCD or Sup in the process of quality examination [74]. Meanwhile, mutual understanding and trust between the CCD and the CMD are still lacking because of the excessively high purchase price or wastage of materials. Therefore, the CMD is a relatively isolated actor.
2. Unlike the progress or cost problems, safety issues in the construction project are too complex to be completely presented by BIM [75]. Safety issues include not only the unsafe behaviours of workers and the overall state of things but also the safety hazards of engineering quality. Thus, the complexity leads to key quality, and safety information cannot be shared fully between the CSD and Sup by a single BIM technology.
3. Traditionally, the project operation plan of the owner does not require many details. OOD tends to rely on experience and uses traditional chart methods to carry out most work rather than BIM. As a result, the acceptance of BIM in the OOD is generally low. Similarly, considering that the subcontractor’s company scale and their work amount are relatively small, CSD is reluctant to increase the upfront investment too much to adapt to the BIM context.

In a word, these marginal participants are still unable to utilise BIM to achieve better consensus and collaboration with others. Thus, some issues still need to be solved in the process of promoting the informatisation of construction project management.

## 6. Implications

### 6.1. Theoretical implications

This research reveals important insights into construction project management and SC theory by focusing on the comprehensive impacts of BIM use on organisational communication of project. At present, research on the simultaneous integration of inter- and intra-organisational network relationships amongst all participants of a construction project is limited, particularly in the case of BIM use. To bridge this gap in the literature, our study introduces the concept of SC, which is closely related to the existing relationships amongst members, to reflect the degrees of communication and collaboration within a relationship network of project. Then, this study explores changes in inter- and intra-organisational network relationships after using BIM in the construction project. To the best of our knowledge, this work is one of the early efforts to investigate communication and collaboration in a construction project team against the background of informatisation of relationships within and amongst organisations. The findings of this work can provide reference for conducting relevant theoretical research in the future.

This research also has implications for identifying conditions and proposing strategies for strengthening organisational collaboration in BIM-enabled network relationships of project. Like prior studies, our research has indicated that BIM use plays an important role in fostering communication and collaboration amongst members of a construction project team. However, a deep discussion for the problems existing in communication networks after BIM use remains lacking. Our research suggests that marginal participants still cannot use BIM to improve their communication and collaboration with other members of the team. Then, the conditions and strategies for strengthening BIM-based organisational communication and collaboration were further identified and proposed. The research findings thus expand the understanding of BIM technology in the field of complex engineering applications. This contribution evidently complements the existing body of knowledge regarding BIM.

### 6.2. Practical implications

This study presents several implications with a reference value for managerial practice. As a construction ICT, BIM is used throughout the lifecycle of a construction project by bringing together the work of various disciplines, to encourage quick and unified information sharing and improve collaboration amongst participants. Thus, managers should pay increasing attention to the role of BIM in team SC. BIM can overcome time and space barriers of communication among different participants and further promote members’ consensus and trust, which encourage organisation members to share their knowledge effectively.

However, managers should understand that there are still some deficiencies in the application of BIM. For example, some tasks are too complex or fragmentary to be presented by a single BIM. As a result, marginal participants are unable to utilise BIM to establish better consensus and collaboration with others.

Therefore, managers can combine multiple ICTs to achieve more adequate identification and sharing of security information. For example, geographic information system (GIS), global positioning system (GPS), computer vision and BIM can be combined to better present the specific location of dangerous parts as well as to achieve personnel and equipment position tracking and hazard warning at any time; Unmanned aerial vehicles (UAVs), augmented reality (AR) and BIM can be combined to identify and monitor potential quality hazards at different locations of a project. RFID technology can also be used to track the information of materials and equipment, such as the type, inventory status, use location and consumed quantity, which can be embedded into the BIM model. A cloud-based BIM platform can be built to store and analyse large information of about the construction project, which can help in achieving real-time interaction and unified understanding amongst participants.

## 7. Conclusions

This study determines the communication network relationships before and after the use of BIM in a project in China from the dimensions of SC and measures the changes of network metrics by SNA. The results show that, compared with the situation before BIM application, the connections between network nodes are closer after BIM application. Thus, participants in the construction project team have more interaction and consensus basis. Furthermore, all the values of the network centralisation decreased, suggesting that the information distribution in the communication network became more balanced after BIM application. Nevertheless, the results also show that some construction project participants with interaction and communication needs remained unable to establish effective connections with others even after BIM use. Therefore, these marginal participants are still unable to utilise BIM to foster better consensus and collaboration with others. Thus, some issues still need to be solved in the process of promoting the informatisation of construction project management.

This research contributes to (a) the state of knowledge by proposing social capital perspective that can identify inter- and intra-organizational relationships of the construction project from social interaction and common cognition to build communication network and (b) the state of practice by identifying conditions and proposing strategies for strengthening organisational communication and collaboration in BIM-enabled network relationships.

Apart from the findings, some limitations of this study need to be discussed, along with future research directions that need to be explored. One of the limitations of this study is that it focused only on the participants directly involved in the construction project implementation. Other stakeholders who may have had an indirect impact on the project were excluded. The upgraded BIM may further blur the organisational boundaries and flatten the organisational structure. Therefore, future studies can further analyse the network relationships among the general or expanded stakeholders of a construction project under BIM influence.

Moreover, this study, as an illustrative example, is probably not the most typical representative of the broader construction industry. The generalisability of this study’s results is restricted by the sample size (one project case) and geographical location (China). Other construction projects may be implemented with various organisational structures and cooperative atmospheres. Therefore, more case studies are needed to make these findings universal.

Finally, SC theory contends that social relationships are resources that can lead to the development and accumulation of human capital. Thus, the given method has no discussion on potential downsides, (e.g., acting as a barrier to social inclusion and social mobility, dividing rather than uniting communities or societies, and so on). Therefore, future studies can attempt to further explore the issues.

## Supporting information

S1 File Outline of interview questions file. (DOCX)

S2 File Interview data file. (XLSX)

S3 File Consent letter file. (DOCX)

S4 File Anonymous interviewees. (XLSX)

S5 File EFA. (SAV)

S1 Appendix The list of abbreviation. (DOCX)
