# Supplementary material for: Impacts of building information modelling (BIM) on communication network of the construction project: A social capital perspective
Source: PLoS One. 2022 Oct 11;17(10):e0275833. doi: 10.1371/journal.pone.0275833 (PMC9553046; doi:10.1371/journal.pone.0275833)
Supplement: S1 File — (DOCX) [file pone.0275833.s001.docx]

Dear Expert:

In order to reveal the comprehensive impacts of building information modelling (BIM) use on organisational communication of construction project, we conduct this interview. The interview requires you to answer which participants you may have social interactions with and shared cognitions with.

| No. | Questions description | Responses of interviewees (mark the mentioned participants “√”) | | | | | | | | | | | | | |
| --- | --- | --- | --- | --- | --- | --- | --- | --- | --- | --- | --- | --- | --- | --- | --- |
| 1 | Social interaction | OED | ODD | OOD | OCD | Des | Sup | CED | CTD | CCD | CMD | CSD | SED | SCD | BC |
| 1.1 | Which participants do you often have to meet formally with? |  |  |  |  |  |  |  |  |  |  |  |  |  |  |
| 1.2 | Which participants do you often communicate informally with? |  |  |  |  |  |  |  |  |  |  |  |  |  |  |
| 1.3 | Which participants do you have close relationships with? |  |  |  |  |  |  |  |  |  |  |  |  |  |  |
| 1.4 | Which participants have you worked with before starting this project? |  |  |  |  |  |  |  |  |  |  |  |  |  |  |
| 1.5 | Which participants have you worked with to solve construction problems in this project? |  |  |  |  |  |  |  |  |  |  |  |  |  |  |
| 2 | Common cognition |  |  |  |  |  |  |  |  |  |  |  |  |  |  |
| 2.1 | Which participants are you more likely to reach agreement with when talking about important issues? |  |  |  |  |  |  |  |  |  |  |  |  |  |  |
| 2.2 | Which participants share the same vision and ambitions with you? |  |  |  |  |  |  |  |  |  |  |  |  |  |  |
| 2.3 | Which participants’ ideas can you accurately understand? |  |  |  |  |  |  |  |  |  |  |  |  |  |  |
| 2.4 | Which participants use the same terminology as you do when describing the work? |  |  |  |  |  |  |  |  |  |  |  |  |  |  |
| 2.5 | Which participants do you think have the same knowledge background as you? |  |  |  |  |  |  |  |  |  |  |  |  |  |  |

Thank you for taking the time to participate in this interview!

Research Group on BIM use
