# Supplementary material for: Impacts of building information modelling (BIM) on communication network of the construction project: A social capital perspective
Source: PLoS One. 2022 Oct 11;17(10):e0275833. doi: 10.1371/journal.pone.0275833 (PMC9553046; doi:10.1371/journal.pone.0275833)
Supplement: S3 File — (DOCX) [file pone.0275833.s003.docx]

**Interview Consent**

Dear Sir/Madam:

Thank you very much for participating in this interview! We are currently conducting a research on the impact of building information modeling (BIM) on communication network of construction project. The objective of this study is to reveal the comprehensive impacts of BIM use on organisational communication, and to identify conditions and propose strategies for strengthening organisational collaboration in BIM-enabled network relationships.

We guarantee that the interview results and all relevant information obtained in the interview will only be used in academic research. The relevant information of the interviewee is strictly confidential.

Thank you very much for your support!

Research Group on BIM use
