# Supplementary material for: Impacts of building information modelling (BIM) on communication network of the construction project: A social capital perspective
Source: PLoS One. 2022 Oct 11;17(10):e0275833. doi: 10.1371/journal.pone.0275833 (PMC9553046; doi:10.1371/journal.pone.0275833)
Supplement: S1 Appendix — (DOCX) [file pone.0275833.s006.docx]

**Appendix A.** The list of abbreviation

| No. | Full name | Abbreviation |
| --- | --- | --- |
| 1 | building information modelling | BIM |
| 2 | architecture, engineering, and construction | AEC |
| 3 | social capital | SC |
| 4 | social network analysis | SNA |
| 5 | information and communication technology | ICT |
| 6 | cognitive social structure | CSS |
| 7 | geographic information system | GIS |
| 8 | global positioning system | GPS |
| 9 | unmanned aerial vehicles | UAVs |
| 10 | augmented reality | AR |
| 11 | radio frequency identification | RFID |
| 12 | owner’s engineering department | OED |
| 13 | owner’s design department | ODD |
| 14 | owner’s operation department | OOD |
| 15 | owner’s cost department | OCD |
| 16 | designer | Des |
| 17 | supervision | Sup |
| 18 | BIM consultation | BC |
| 19 | contractor’s engineering department | CED |
| 20 | contractor’s technology department | CTD |
| 21 | contractor’s cost department | CCD |
| 22 | contractor’s material department | CMD |
| 23 | contractor’s safety department | CSD |
| 24 | subcontractor’s engineering department | SED |
| 25 | subcontractor’s cost department | SCD |
